# Protection against H5N1 Highly Pathogenic Avian and Pandemic (H1N1) 2009 Influenza Virus Infection in Cynomolgus Monkeys by an Inactivated H5N1 Whole Particle Vaccine

**DOI:** 10.1371/journal.pone.0082740

**Published:** 2013-12-23

**Authors:** Misako Nakayama, Shintaro Shichinohe, Yasushi Itoh, Hirohito Ishigaki, Mitsutaka Kitano, Masahiko Arikata, Van Loi Pham, Hideaki Ishida, Naoko Kitagawa, Masatoshi Okamatsu, Yoshihiro Sakoda, Takaya Ichikawa, Hideaki Tsuchiya, Shinichiro Nakamura, Quynh Mai Le, Mutsumi Ito, Yoshihiro Kawaoka, Hiroshi Kida, Kazumasa Ogasawara

**Affiliations:** 1 Division of Pathology and Disease Regulation, Department of Pathology, Shiga University of Medical Science, Otsu, Japan; 2 Department of Otorhinolaryngology-Head and Neck Surgery, Shiga University of Medical Science, Otsu, Japan; 3 Research Center for Animal Life Science, Shiga University of Medical Science, Otsu, Japan; 4 Laboratory of Microbiology, Department of Disease Control, Graduate School of Veterinary Medicine, Hokkaido University, Sapporo, Japan; 5 Research Center for Zoonosis Control, Hokkaido University, Sapporo, Japan; 6 National Institute of Hygiene and Epidemiology, Hanoi, Vietnam; 7 Division of Virology, Department of Microbiology and Immunology, Institute of Medical Science, University of Tokyo, Tokyo, Japan; 8 Department of Pathobiological Sciences, University of Wisconsin-Madison, Madison, Wisconsin, United States of America; German Primate Center, Germany

## Abstract

H5N1 highly pathogenic avian influenza virus (HPAIV) infection has been reported in poultry and humans with expanding clade designations. Therefore, a vaccine that induces immunity against a broad spectrum of H5N1 viruses is preferable for pandemic preparedness. We established a second H5N1 vaccine candidate, A/duck/Hokkaido/Vac-3/2007 (Vac-3), in our virus library and examined the efficacy of inactivated whole particles of this strain against two clades of H5N1 HPAIV strains that caused severe morbidity in cynomolgus macaques. Virus propagation in vaccinated macaques infected with either of the H5N1 HPAIV strains was prevented compared with that in unvaccinated macaques. This vaccine also prevented propagation of a pandemic (H1N1) 2009 virus in macaques. In the vaccinated macaques, neutralization activity, which was mainly shown by anti-hemagglutinin antibody, against H5N1 HPAIVs in plasma was detected, but that against H1N1 virus was not detected. However, neuraminidase inhibition activity in plasma and T-lymphocyte responses in lymph nodes against H1N1 virus were detected. Therefore, cross-clade and heterosubtypic protective immunity in macaques consisted of humoral and cellular immunity induced by vaccination with Vac-3.

## Introduction

H5N1 highly pathogenic avian influenza virus (HPAIV) infection in humans has been reported since 1997 (http://www.who.int/influenza/human_animal_interface/H5N1_cumulative_table_archives/en/). Although H5N1 HPAIVs did not appear to transmit easily among humans (http://www.who.int/influenza/human_animal_interface/Influenza_Summary_IRA_HA_interface_04Jun13.pdf), the public health risks associated with H5N1 HPAIVs remain unchanged since most humans do not possess immunity against H5N1 virus and H5N1 HPAIVs have been detected in poultry and swine [1], [2], of which the latter is thought to be an origin of past pandemic virus [3]–[5]. Therefore, development of vaccines against H5N1 HPAIVs has been required.

Mutation rates in hemagglutinin (HA) genes of avian and swine influenza viruses were lower than those in HA genes of human seasonal influenza viruses [1], [6]. However, H5N1 HPAIVs have genetically been divided into several clades according to HA sequences, and further evolution of the virus has led to the appearance of new clades and subclades as of 2012 [7], [8]. Therefore, it is thought that vaccine strains should be renewed according to circulating strains, and the development of a vaccine that is effective against a broad spectrum of different clades is required [9].

We have established a vaccine library containing 144 different subtypes of non- or low pathogenic influenza viruses with combinations of 16 hemagglutinins (HA) and 9 neuraminidases (NA) [10]. We previously selected vaccine candidate strains from the library to examine their efficacy against H5N1, H7N7, and H1N1 virus infections in cynomolgus macaques [11]–[13]. To update vaccine candidates, we developed a second strain of H5N1 subtype low pathogenic reassortant influenza virus, A/duck/Hokkaido/Vac-3/2007 (Vac-3) [14]. The Vac-3 virus propagated more vigorously in embryonated eggs than did Vac-1, which was the first non-pathogenic H5N1 virus in the virus library [11]. Therefore, if Vac-3 induced protective immunity against H5N1 HPAIVs, it would be a suitable vaccine candidate for vaccine production to reduce the number of embryonated eggs required and to produce vaccines more rapidly at pandemics [15].

In the present study, immunogenicity of the Vac-3 vaccine and its protective efficacy against two H5N1 HPAIVs in different clades in macaques were analyzed. Whole virus particles of Vac-3 inactivated by formalin were subcutaneously inoculated into macaques. Neutralization activity of plasma against the vaccine strain was detected in all macaques. In challenge infections, duration of virus detection in vaccinated macaques infected with the two different clades of H5N1 HPAIVs was shorter than that of virus detection in unvaccinated macaques. Furthermore, propagation of a pandemic (H1N1) 2009 virus in macaques vaccinated with Vac-3 was prevented. The protection of vaccinated macaques from H5N1 HPAIV and pandemic (H1N1) 2009 virus infection was due to antibody responses against HA and NA and to T lymphocyte responses against viral antigens. Thus, the whole particle vaccine of Vac-3 induced immune responses against multiple clades and subtypes.

## Results

### Pathogenicity of Two H5N1 Highly Pathogenic Avian Influenza Virus Strains in Cynomolgus Macaques

**Figure 1:**
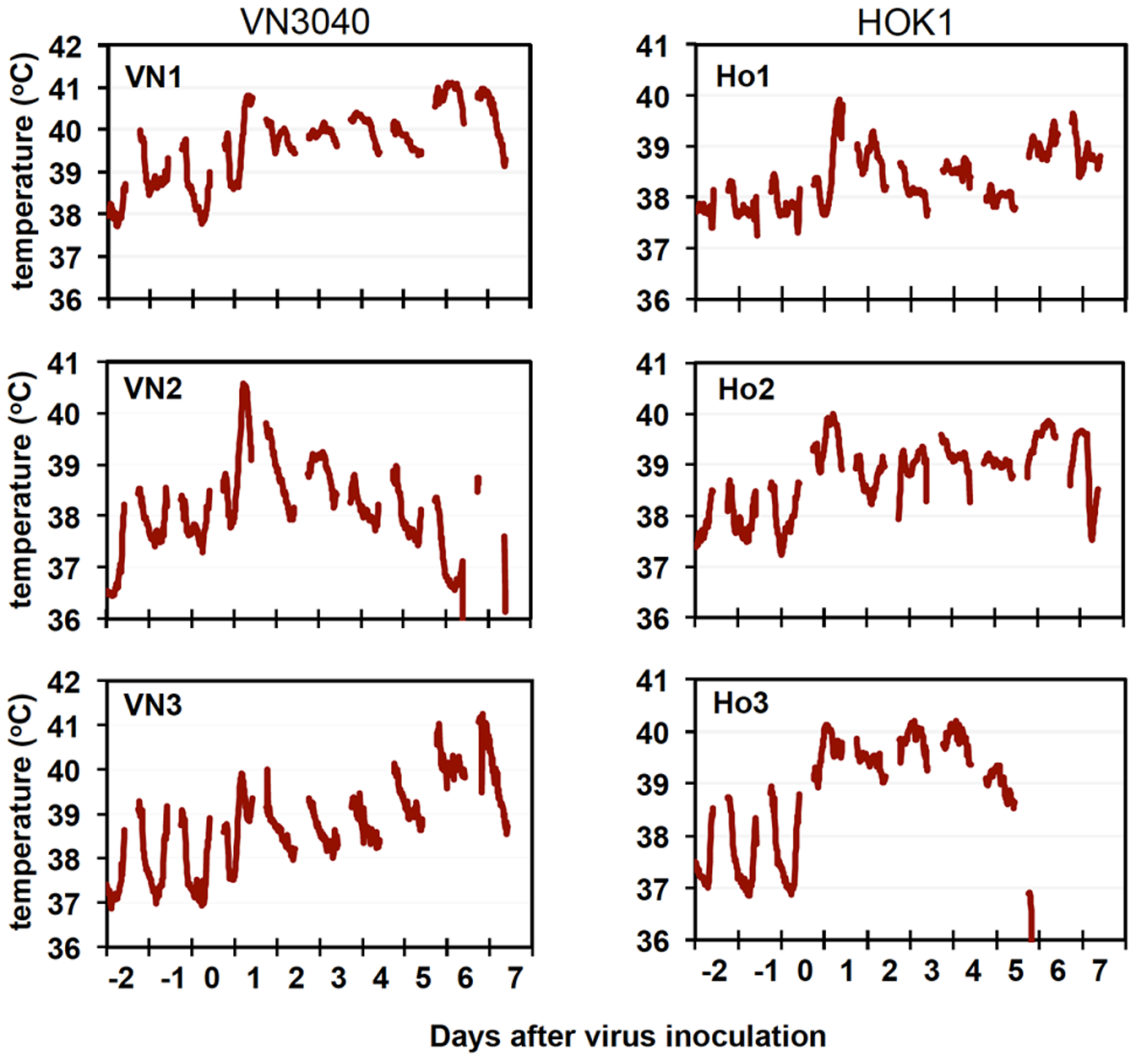
Body temperature of cynomolgus macaques infected with H5N1 highly pathogenic avian influenza virus. Highly pathogenic avian influenza virus A/Vietnam/UT3040/2004 (H5N1) (left, VN3040) or A/whooper swan/Hokkaido/1/2008 (H5N1) (right, HOK1) was inoculated into the nostrils, oral cavity, and trachea of each macaque on day 0. Body temperature of macaques was recorded using telemetry transmitters and a computer. Temperatures from 6 P.M. to 10 A.M. are shown in the graphs since temperatures between 10 A.M. and 6 P.M. were affected by anesthesia.

Firstly, we examined the pathogenicity of highly pathogenic avian influenza viruses, A/Vietnam/UT3040/2004 (H5N1) (clade 1, VN3040) and A/whooper swan/Hokkaido/1/2008 (H5N1) (clade 2.3.2.1, HOK1), in cynomolgus macaques. After inoculation of the virus into nasal cavities, oral cavities, and tracheas, all macaques infected with either virus showed higher body temperatures over 40°C than those before infection (Figure 1). The average of clinical scores diagnosed according to Table S4 in macaques inoculated with HOK1 was higher than that in macaques inoculated with VN3040 although the difference was not statistically significant (Figure S1). One of the macaques inoculated with HOK1, named Ho3 (abbreviations indicated in Table S1), died 5 days after infection (survival rates on day 7 was 3/3 and 2/3 in macaques inoculated with VN3040 and HOK1, respectively). The viruses were recovered from nasal, oral, tracheal, and bronchial samples from macaques infected with either strain until days 6 to 7 (Tables 1 and 2). Therefore, both viruses propagated in upper and lower respiratory tracts of macaques.

**Table 1:** Virus titers in swab samples from cynomolgus macaques infected with H5N1 highly pathogenic avian influenza virus, VN3040.

			Virus titers (log_10_TCID_50_/ml)						
			Days after infection						
Sample	Vaccine	Animal	1	2	3	4	5	6	7
Nasal cavity	−	VN1	2.45	2.45	1.75	2.56	1.40	<*	<
	−	VN2	5.36	3.84	2.80	3.84	3.26	3.15	2.95
	−	VN3	<	2.10	1.63	4.66	5.21	5.39	3.84
	+	VN4	2.00	<	<	<	<	<	<
	+	VN5	2.50	1.77	1.50	1.23	<	<	<
	+	VN6	<	<	<	<	<	<	<
Oral cavity	−	VN1	<	3.15	2.33	<	<	1.92	<
	−	VN2	3.03	2.10	1.63	1.86	1.94	<*	<
	−	VN3	<	2.10	2.68	2.56	2.56	2.80	<
	+	VN4	2.50	<	<	<	<	<	<
	+	VN5	2.77	2.00	<	<	1.83	<	<
	+	VN6	<	<	<	<	<	<	<
Trachea	−	VN1	5.21	5.77	3.84	2.80	3.66	4.03	2.21
	−	VN2	3.84	2.56	1.75	<	2.26	1.63	<
	−	VN3	4.43	3.84	3.26	3.66	3.03	3.15	<
	+	VN4	2.33	<	<	<	<	<	<
	+	VN5	1.50	1.50	<	2.33	<	<	<
	+	VN6	<*	<	<	<	<	<	<
Bronchus	−	VN1	5.13	3.84	4.43	3.38	4.51	3.03	3.96
	−	VN2	3.84	2.10	2.63	<	<	<	<
	−	VN3	4.19	3.15	3.66	2.10	1.56	<*	<
	+	VN4	2.23	<*	<	<	<	<	<
	+	VN5	2.50	1.33	<	<	<	<	<
	+	VN6	<	<	<	<	<	<	<

_50_/ml). <: Virus titers were under the detection limit (0.67 TCID

*: One CPE positive well was observed in quadruplicate culture of undiluted samples. <

**Table 2:** Virus titers in swab samples from cynomolgus macaques infected with H5N1 highly pathogenic avian influenza virus, HOK1.

			Virus titers (log_10_TCID_50_/ml)						
			Days after infection						
Sample	Vaccine	Animal	1	2	3	4	5	6	7
Nasal cavity	−	Ho1	1.50	<1	<	<*	<1	2.00	<1.3
	−	Ho2	4.50	2.23	2.50	2.50	2.67	2.50	2.50
	−	Ho3	3.70	<	3.67	2.33	3.50	NT	NT
	+	Ho4	<	<	<	<	<	<	<
	+	Ho5	<	<	<	<	<	<	<
	+	Ho6	<	1.33	<	<	<	<	<
Oral cavity	−	Ho1	2.50	1.50	1.50	<	<	1.33	2.50
	−	Ho2	5.00	2.33	1.50	1.67	2.50	2.33	2.33
	−	Ho3	2.00	<	2.00	3.33	1.33	NT	NT
	+	Ho4	<	<	<	<	<	<	<
	+	Ho5	<	<	<	<	<	<	<
	+	Ho6	<	1.67	<	<	<	<	<
Trachea	−	Ho1	4.50	4.00	3.23	2.50	2.50	2.33	2.67
	−	Ho2	5.67	3.67	2.33	4.00	3.33	3.50	2.67
	−	Ho3	4.50	3.33	2.50	3.33	2.50	NT	NT
	+	Ho4	1.33	<	<	<	<	<	<
	+	Ho5	1.50	<	<	<	<	<	<
	+	Ho6	<*	1.33	<	<	<	<	<
Bronchus	−	Ho1	4.00	2.67	1.50	<1	1.33	<1	<
	−	Ho2	4.67	3.50	2.50	2.00	3.00	<	2.67
	−	Ho3	4.33	2.67	1.50	1.50	2.67	NT	NT
	+	Ho4	1.50	<	<	<	<	<	<
	+	Ho5	3.50	<	<	<	<	<	<
	+	Ho6	3.00	<	<	<	<	<	<

_50_/ml). <: Virus titers were under the detection limit (0.67 TCID

*: One CPE positive well was observed in quadruplicate culture of undiluted samples. NT: not tested. <

We examined virus titers in tissues obtained at autopsy 7 days or 5 days after infection. Virus was detected in the lungs of two and three macaques infected with VN3040 and HOK1, respectively (Table S2). Virus was further detected in the tonsils of all macaques, in the jejunum of one macaque infected with VN3040 (VN2), and in samples of the brain and muscle in a dead macaque infected with HOK1 (Ho3). Therefore, these two H5N1 strains propagated not only in upper and lower respiratory tracts but also in non-respiratory organs of macaques.

**Figure 2:**
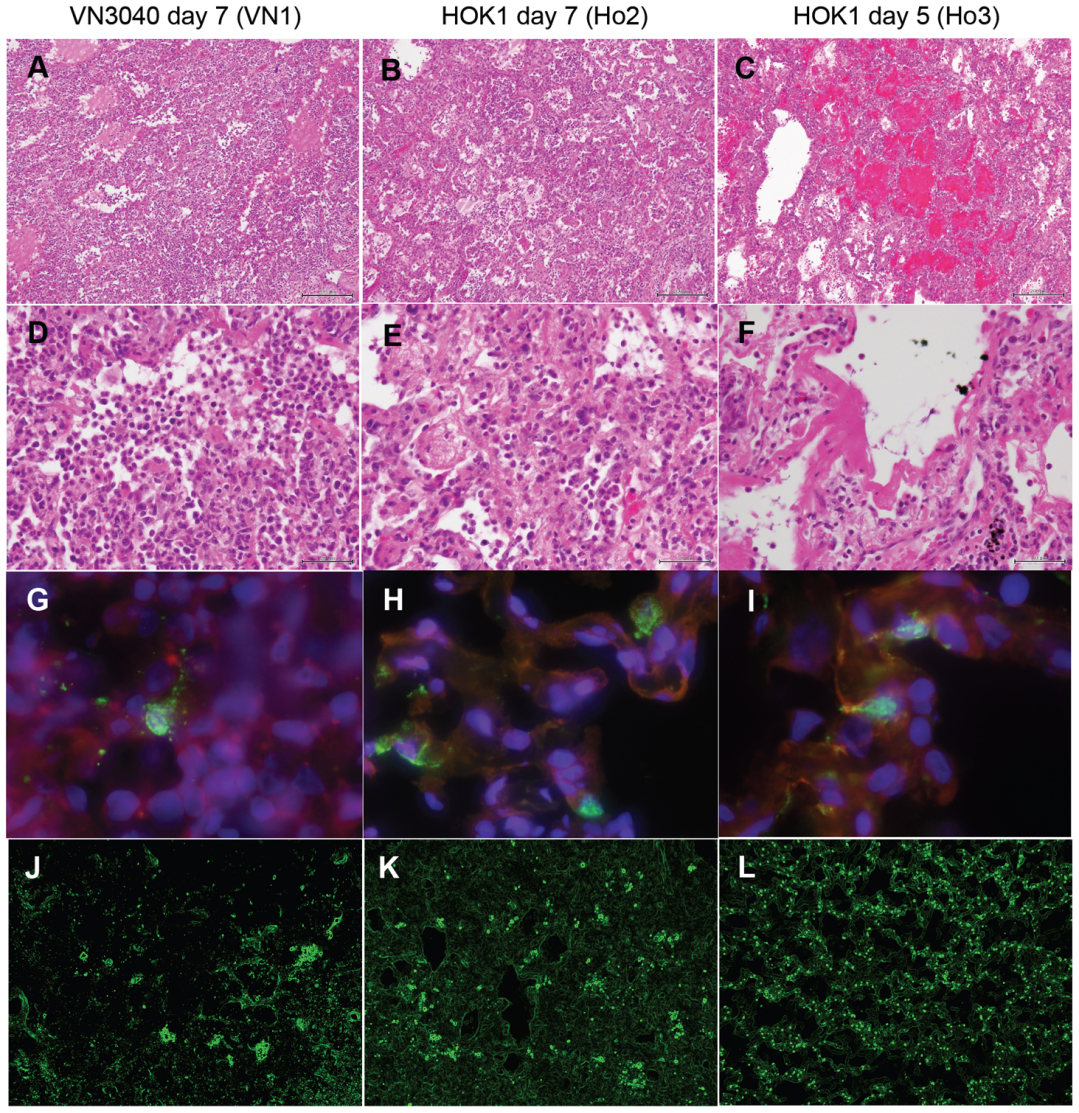
Viral pneumonia in macaques infected with H5N1 highly pathogenic avian influenza virus. One macaque, VN1 (A, D, G, J), was inoculated with VN3040. Two macaques, Ho2 (B, E, H, K) and Ho3 (C, F, I, L), were inoculated with HOK1. VN1 and Ho2 were autopsied 7 days after virus inoculation. The dead macaque Ho3 was autopsied 5 days after virus infection. (A–F) Lung tissue sections were stained with H & E. A–C: low magnification (bars: 200 µm), D–F: high magnification (bars: 50 µm). (G–I) Viral antigen was stained with FITC-conjugated anti-influenza A virus antibody (green) in combination with eFluor615-conjugated cytokeratin (red) and DAPI for staining of nuclei (blue). (J–L) The distribution of viral antigen in lungs stained with FITC-conjugated anti-influenza A virus antibody (bright green). The images were analyzed under a sobel edge detection filter.

Histological changes in lungs of macaques infected with VN3040 and HOK1 were assessed. Capacity for air exchange was reduced in alveoli of macaques 7 and 5 days after infection with VN3040 and HOK1 (Figure 2A–C). Alveolar septa were thickened with inflammatory cells and fibrinous exudate, accompanied by infiltration of predominantly lymphocytes in the alveoli (Figure 2D–F). These pathological results suggested impaired respiratory function in the macaques infected with H5N1 HPAIVs. In the macaque that died on day 5 (Ho3), hemorrhage and lymphocyte infiltration in alveolar spaces were observed (Figure 2C). Formation of hyaline membranes was observed in part, suggesting that diffuse alveolar damage and acute respiratory distress syndrome occurred (Figure 2F). In each macaque, influenza A virus antigen (green) was detected by immunofluorescence staining mainly in the nuclei (bright green after merging) and partially in the cytoplasm (yellow after merging) of type II pneumocytes that were cuboidal alveolar lining cells positive for pan-cytokeratin (red) (Figure 2G–I). The distribution of viral antigen-positive cells was focal and patchy in the lungs of the macaques infected with VN3040, whereas the distribution was diffuse in the lungs of macaques infected with HOK1 (Figure 2J, K). Especially in Ho3, viral antigen-positive cells were observed in every alveolus, which correlated with the severity of pneumonia (Figure 2L). Although HOK1 showed higher pathogenicity in cynomolgus macaques than did VN3040, both H5N1 HPAIVs used in the present study induced severe morbidity with viral pneumonia in cynomolgus macaques and that they were considered suitable for evaluation of vaccine efficacy.

### Antibody Responses in Macaques after Vaccination with Vac-3

To examine the immunogenicity of Vac-3 in macaques, we inoculated twelve macaques with inactivated whole viral particles of Vac-3 subcutaneously twice with a two-week interval without addition of an adjuvant since inactivated whole virus particles included viral RNA as an internal adjuvant and the interval was sufficient to prime and boost immune responses within a month from initiation of vaccination in our previous studies using macaques [12], [13], [16]. Two weeks after the first vaccination (before the second vaccination), IgG antibody specific for Vac-3 antigen was detected in plasma, nasal swab samples, and tracheal swab samples from all vaccinated macaques (Figure S2A–C). Two weeks after the second vaccination (four weeks after the first vaccination), levels of IgG specific for Vac-3 antigen were increased in all macaques. No production of IgA specific for Vac-3 antigen was detected in plasma (Figure S2D). On the other hand, IgA production was detected in nasal swab samples from all macaques except NL4 and in tracheal samples from only one macaque, NL5 (Figure S2E, F). Plasma collected from all vaccinated macaques 2 weeks after the second vaccination (week 4) showed neutralization activity against the vaccine strain, Vac-3 (Table 3). Thus, the whole particle vaccine of Vac-3 was immunogenic in cynomolgus macaques.

**Table 3:** Neutralization activity against the vaccine strain Vac-3 in plasma.

		Weeks after 1^st^ vaccination			Days after challenge infection				
Challenge	Animal	0	2	4	0	1	3	5	7
VN3040	VN4	<2	4.7	7.2	5.9	5.6	6.5	7.0	8.1
	VN5	<2	2.6	4.4	5.6	7.5	7.1	6.9	10.1
	VN6	<2	4.6	7.5	7.6	10.0	9.6	11.1	>12
HOK1	Ho4	<4	<2	5.5	6.9	8.5	9.3	10.4	11.4
	Ho5	<2	2.3	9.1	7.9	9.1	5.3	9.0	8.8
	Ho6	<2	2.6	5.8	5.6	4.8	5.5	6.3	8.8
NL2586	NL4	<2	<2	3.8	4.5	4.6	4.8	4.6	4.6
	NL5	<2	7.9	10.8	11.4	11.6	10.9	8.8	NT
	NL6	<2	6.2	11.1	8.7	5.9	9.1	9.6	9.6
NRT1	NR4	<2	<2	4.6	7.9	5.7	6.1	6.3	6.3
	NR5	<2	3.1	8.0	7.8	6.6	6.9	5.0	5.2
	NR6	<2	2.7	6.9	9.0	9.1	9.1	8.8	9.1

% neutralization activity are indicated as log_2_ dilution. NT: not tested. The interval between 4 weeks after the first vaccination and day 0 (day of infection) was 3 weeks. Dilution titers that show 50

### Challenge Infection of Macaques Immunized with Vac-3

**Figure 3:**
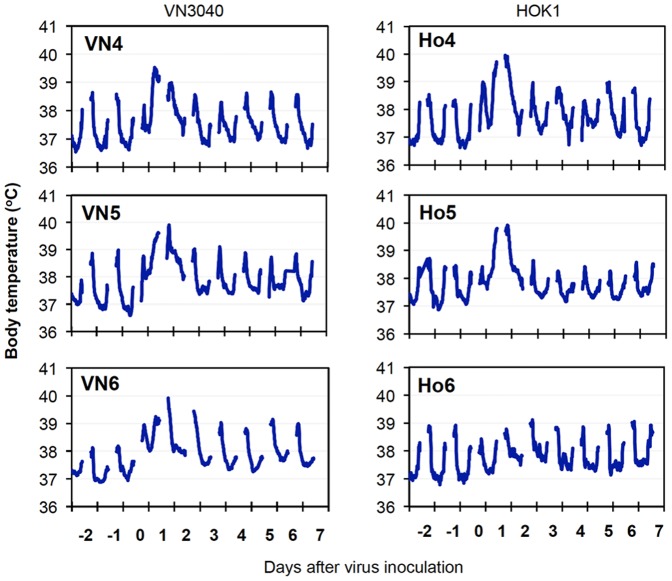
Body temperature of vaccinated macaques infected with H5N1 highly pathogenic avian influenza virus. H5N1 highly pathogenic avian influenza virus VN3040 (left) or HOK1 (right) was inoculated into the nostrils, oral cavity, and trachea of macaques on day 0 (five weeks after the second vaccination). Body temperatures of macaques were recorded using telemetry transmitters and a computer. Temperatures from 6 P.M. to 10 A.M. are shown in the graphs since temperatures between 10 A.M. and 6 P.M. were affected by anesthesia.

To examine the efficacy of vaccination with Vac-3, challenge virus strains VN3040 and HOK1, which have similarity of amino acid sequences to Vac-3 between 89% and 93% in HA and NA (Table S3), were inoculated into the vaccinated macaques. All macaques infected with VN3040 and two macaques infected with HOK1 (Ho4 and Ho5) showed higher body temperatures after infection than those before virus infection (Figure 3). The other macaque infected with HOK1 (Ho6) showed a rise in body temperature at night. The high body temperatures returned to normal levels on day 3 after infection and all of the macaques survived until day 7, at which point the study was terminated for tissue collection. Clinical scores of the vaccinated macaques infected with HOK1 were significantly lower than those of unvaccinated macaques 2 to 5 days after virus infection (Figure S1B). Virus was detected in oral samples from the macaques inoculated with VN3040 until day 5 and in nasal, oral, and tracheal samples from macaques inoculated with HOK1 until day 2 after infection (Tables 1 and 2). The durations of virus detection in the vaccinated macaques were shorter than those in the unvaccinated macaques. Virus titer areas under the virus titer time curves (virus titer AUCs) in the samples from vaccinated macaques were significantly lower than those in samples from unvaccinated macaques (Figure S1E, F). No virus was detected in tissue samples obtained at autopsy on day 7 after infection (data not shown). Therefore, vaccination with whole viral particles of Vac-3 inhibited H5N1 HPAIV propagation in macaques and reduced morbidity.

Next, a different subtype of HPAIV, A/chicken/Netherlands/2586/2003 (H7N7) (NL2586), or a pandemic (H1N1) 2009 virus, A/Narita/1/2009 (H1N1) (NRT1), was inoculated into macaques vaccinated with Vac-3 to examine crossreactivity of immunity induced by Vac-3. As for the unvaccinated macaques infected with NL2586 or NRT1, some data reported previously were cited for the comparison with the vaccinated macaques [16], [17]. The vaccinated macaques showed higher body temperatures after infection with NL2586 than those before infection (Figure S3, upper right), as seen in the unvaccinated macaques in the previous study (Figure S3, upper left) [16]. No significant improvement in the clinical scores of the vaccinated macaques was seen compared with the unvaccinated macaques (Figure S1C). One of the macaques (NL5) vaccinated with Vac-3 died on day 4 after NL2586 virus infection. Virus in nasal, oral, tracheal, and bronchial samples from the vaccinated macaques was detected until day 6, day 5, day 7 and day 4 after virus infection, respectively (Table 4). On the other hand, in the unvaccinated macaques, of which virus titers in nasal samples were previously reported [16], virus in nasal, oral, tracheal, and bronchial samples was detected until day 6, day 7, day 7 and day 6, respectively. No significant differences in the virus titer AUCs of the vaccinated and unvaccinated macaques until day 4 were seen (Figure S1G). Therefore, vaccination with Vac-3 had little effect on protection against H7N7 virus infection, though durations of virus detection in oral and bronchial samples from the vaccinated macaques were two days shorter than those of virus detection in oral and bronchial samples from the unvaccinated macaques.

**Table 4:** Virus titers in swab samples from cynomolgus macaques infected with NL2586.

			Virus titers (log_10_ TCID_50_/ml)						
			Days after infection						
Sample	Vaccine	Animal	1	2	3	4	5	6	7
Nasal cavity	−	NL1	3.50	3.67	3.00	5.33	3.50	2.67	<
	−	NL2	2.67	2.33	2.00	2.23	<	<*	<
	−	NL3	<*	<	<*	1.67	1.67	2.50	<
	+	NL4	4.50	3.33	3.50	2.50	2.23	1.50	<
	+	NL5	4.67	3.67	2.50	2.23	NT	NT	NT
	+	NL6	4.50	2.67	2.67	1.67	2.33	<	<
Oral cavity	−	NL1	2.33	1.67	1.50	<*	2.33	<*	1.50
	−	NL2	2.00	2.33	<*	<	<	<	<*
	−	NL3	2.67	<*	<	<	2.00	2.50	<*
	+	NL4	4.67	3.00	2.17	1.67	<	<	<
	+	NL5	1.67	2.50	1.50	2.33	NT	NT	NT
	+	NL6	4.33	<	1.23	<*	1.67	<	<
Trachea	−	NL1	3.33	3.33	<*	3.00	3.33	2.83	<
	−	NL2	4.23	2.00	<*	<*	<*	<*	<*
	−	NL3	2.67	1.67	<	<*	2.00	3.00	<
	+	NL4	4.50	3.77	1.33	2.00	2.50	2.33	<*
	+	NL5	3.50	3.23	1.50	2.33	NT	NT	NT
	+	NL6	5.33	2.67	1.83	<	0.83	<	<
Bronchus	−	NL1	3.50	3.67	2.50	1.67	4.33	1.50	<
	−	NL2	3.67	2.50	1.50	<*	<*	<	<
	−	NL3	3.50	3.67	1.50	<	2.67	<	<
	+	NL4	3.67	3.77	<	<	<	<	<
	+	NL5	3.67	2.67	<	1.33	NT	NT	NT
	+	NL6	4.50	3.23	<	<	<	<	<

_50_/ml). <: Virus titers were under the detection limit (0.67 TCID

*: One CPE positive well was observed in quadruplicate culture of undiluted samples. NT: not tested. Virus titers in nasal samples from unvaccinated macaques were referred from a reference 16. <

In the vaccinated macaques infected with NRT1, although clinical scores were not significantly different between vaccinated and unvaccinated macaques (Figure S1D), increase in body temperature was not as great as increases in body temperatures of macaques infected with HPAIVs and unvaccinated macaques previously reported (Figure S3, lower graphs) [17]. A low level of virus was detected in samples from one macaque (NR4) compared with virus in unvaccinated macaques previously reported [17] (Table 5). No virus was detected in samples from two other macaques (NR5 and NR6). Therefore, virus titer AUCs of the vaccinated macaques were significantly lower than those of the unvaccinated macaques (Figure S1H). Thus, vaccination with Vac-3 induced protective immunity against the pandemic (H1N1) 2009 virus NRT1.

**Table 5:** Virus titers in swab samples from cynomolgus macaques infected with NRT1.

			Virus titers (log_10_TCID_50_/ml)						
			Days after infection						
Sample	Vaccine	Animal	1	2	3	4	5	6	7
Nasal cavity	−	NR1	<	3.49	3.15	5.21	4.69	3.15	<
	−	NR2	<	3.03	2.80	2.21	5.94	4.51	2.26
	−	NR3	4.54	4.51	5.36	5.36	4.66	3.84	<
	+	NR4	1.23	<	<	<	<	<	<
	+	NR5	<	<	<	<	<	<	<
	+	NR6	<	<	<	<	<	<	<
Oral cavity	−	NR1	<	<	<	2.56	3.03	<	<
	−	NR2	<	<	<	2.45	<	1.75	<
	−	NR3	3.49	<	<	<	1.63	3.26	<
	+	NR4	<	<	<	<	<	<	<
	+	NR5	<	<	<	<	<	<	<
	+	NR6	<	<	<	<	<	<	<
Trachea	−	NR1	<	<	<	3.84	4.51	3.96	<
	−	NR2	<	<	<	1.94	1.56	2.80	3.13
	−	NR3	3.84	3.49	3.96	4.19	5.59	3.84	<
	+	NR4	<	<	<	<	<	<	<
	+	NR5	<	<	<	<	<	<	<
	+	NR6	<	<	<	<	<	<	<
Bronchus	−	NR1	<	<	<	3.03	5.05	4.03	<
	−	NR2	<	<	<	<*	<*	2.80	<
	−	NR3	2.40	<*	1.40	<	1.40	<	2.10
	+	NR4	<	<	1.23	<	<	<	<
	+	NR5	<	<	<	<	<	<	<
	+	NR6	<	<	<	<	<	<	<

_50_/ml). <: Virus titers were under the detection limit (0.67 TCID

*: One CPE positive well was observed in quadruplicate culture of undiluted samples. Virus titers in nasal, tracheal, and bronchial samples from unvaccinated macaques were referred from a reference 17. <

### Antibody Responses after Challenge Infection in Macaques Immunized with Vac-3

Immune responses after challenge infection were determined to examine the contribution to protection by humoral and cellular immunity, especially immunity against H1N1 virus, since vaccination with Vac-3 inhibited virus propagation in macaques infected with heterosubtypic NRT1. Neutralization activity against Vac-3 in plasma of macaques infected with VN3040 or HOK1 was increased one to five days after challenge infection (Table 3). The vaccinated macaques challenged by H5N1 HPAIVs showed increase in neutralization activity in plasma against VN3040 or HOK1 five to seven days after challenge infection, though no neutralization activity against challenge viruses was detected before infection (Table 6). The responses seen in the vaccinated macaques were recall memory responses generated by vaccination and reactivated by challenge infection since no neutralization activity against VN3040 or HOK1 was detected in plasma collected on day 7 after infection from the unvaccinated macaques (Table 6).

**Table 6:** Neutralization activity against challenge strains in plasma of macaques.

			Weeks after 1^st^ vaccination		Days after infection		
Challenge virus	Vaccine	Animal	2	4	0	5	7
VN3040	−	VN1	NT	NT	<2	NT	<2
	−	VN2	NT	NT	<2	NT	<2
	−	VN3	NT	NT	<2	NT	<2
	+	VN4	<2	<2	<2	<2	4.0
	+	VN5	<2	<2	<2	<2	3.3
	+	VN6	<2	<2	<2	2.7	6.0
HOK1	−	Ho1	NT	NT	<2	NT	<2
	−	Ho2	NT	NT	<2	NT	<2
	−	Ho3	NT	NT	<2	NT	<2
	+	Ho4	<2	<2	<2	<2	4.0
	+	Ho5	<2	2.0	<2	2.3	5.5
	+	Ho6	<2	<2	<2	<2	3.7
NL2586	−	NL1	NT	NT	<2	<2	<2
	−	NL2	NT	NT	<2	<2	<2
	−	NL3	NT	NT	<2	<2	<2
	+	NL4	<2	<2	<2	<2	<2
	+	NL5	<2	<2	<2	<2	NT
	+	NL6	<2	<2	<2	<2	<2
NRT1	−	NR1	NT	NT	<2	NT	<2
	−	NR2	NT	NT	<2	NT	<2
	−	NR3	NT	NT	<2	NT	<2
	+	NR4	<2	<2	<2	<2	<2
	+	NR5	<2	<2	<2	<2	<2
	+	NR6	<2	<2	<2	<2	<2

% neutralization titers are indicated as log_2_ dilution. NT: not tested. The interval between 4 weeks after the first vaccination and day 0 (day of infection) was 3 weeks. 50

No neutralization activity against NL2586 or NRT1 was detected in plasma collected 7 days after challenge infection in the unvaccinated and vaccinated macaques (Table 6). Furthermore, no increase in neutralization activity against Vac-3 in plasma was seen on day 7 after challenge infection (Table 3). Therefore, memory neutralization antibody responses were not reactivated by challenge infection with NL2586 or NRT1. Furthermore, antibody induced by vaccination with Vac-3 did not show crossreactivity against NL2586 since virus titers in samples from the vaccinated macaques were not different from those in samples from the unvaccinated macaques (Table 4).

We examined anti-NA antibody using neuraminidase inhibition (NI) assay since anti-HA antibody was mainly detected by the neutralization test [18]. Vaccination with Vac-3 induced NI activity against NA of Vac-3 in plasma of the vaccinated macaques two weeks after the second vaccination (Table 7). The same samples also showed NI activity against NA of NRT1. Seven days after challenge infection with NRT1, macaques without vaccination showed low NI titers against NRT1 but not against Vac-3 in plasma. On the other hand, plasma of two macaques vaccinated with Vac-3 (NR4 and NR5) on day 7 showed NI activity against Vac-3 and NRT1 with higher titers than that of the macaques before challenge infection (day 0). These results indicated that anti-NA antibody induced by the Vac-3 vaccine reacted with NA of NRT1 and that the antibody responses were reactivated by challenge infection with NRT1 as memory recall responses.

**Table 7:** Neuraminidase inhibition activity against Vac-3 and NRT1 in plasma of macaques infected with NRT1.

			Weeks after 1^st^ vaccination		Days after challenge infection	
NA source	Vaccine	Animal	0	4	0	7
Vac-3	−	NR1	NT	NT	<	<
	−	NR2	NT	NT	<	<
	−	NR3	NT	NT	<	<
	+	NR4	<	16	16	64
	+	NR5	<	16	16	16
	+	NR6	<	4	16	16
NRT1	−	NR1	NT	NT	<	1
	−	NR2	NT	NT	<	4
	−	NR3	NT	NT	<	4
	+	NR4	<	4	4	64
	+	NR5	<	4	1	64
	+	NR6	<	4	16	16

= undiluted samples). NT: not tested. <: NI activity was under the detection limit (1

### T lymphocyte Responses after Challenge Infection in Macaques Immunized with Vac-3

**Figure 4:**
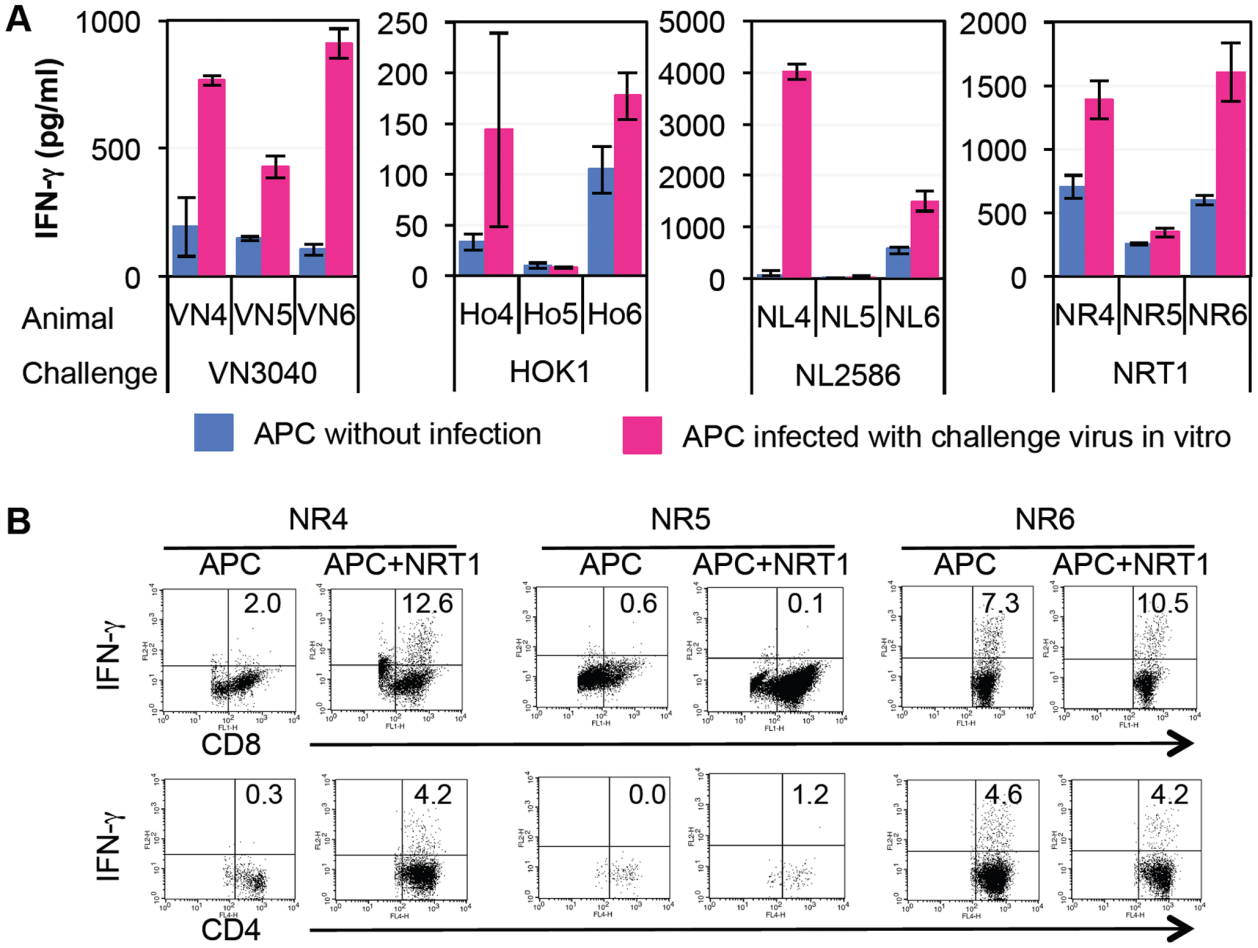
T lymphocyte responses against B cell lines infected with challenge virus. (A) Cervical lymph node cells of vaccinated macaques were isolated 7 or 4 days after challenge infection. The cells were cultured with autologous B cell lines as antigen-presenting cells (APC) with or without challenge virus infection. Two days later, culture supernatants were collected and concentrations of IFN-γ were measured using a bead array assay. Averages and standard deviations of triplicate cultures are shown. Significant differences between APC and APC infected with challenge virus (P<0.05, Student’s t-test) were detected except for Ho4 and Ho5. (B) Cervical lymph node cells from macaques infected with NRT1 were cultured with autologous B cell lines with or without NRT1 infection in the presence of IL-2. Five days later, surviving cells were stimulated with PMA and ionomycin for 5 h. Surface CD8 and CD4 and intracellular IFN-γ were stained and analyzed using a flow cytometer. The percentages of IFN-γ-positive cells in CD4^+^ and CD8^+^ cells are indicated.

To examine T cell responses in vaccinated macaques, we cultured cervical lymph node cells with autologous B cell lines infected with challenge virus as antigen-presenting cells (APC) since cervical lymph nodes were larger and easier to detect than mediastinal lymph nodes at autopsy. Although interferon (IFN)-γ production by lymphocytes was detected in the supernatant cultured with B cell lines without virus infection (Figure 4A), IFN-γ production by lymphocytes obtained from the vaccinated macaques except Ho4 and Ho5 was significantly up-regulated in the presence of APC infected with challenge virus (P<0.05). These results indicated that macaques vaccinated with Vac-3 carried lymphocytes responding to challenge virus antigens on day 7 after challenge infection.

Finally, we examined cells producing IFN-γ in the vaccinated macaques. Cervical lymph node cells were cultured with autologous B cell lines with or without challenge virus infection for 5 days to expand responding cells, and then the cells were restimulated to determine the percentage of IFN-γ-producing cells [13]. Since we did not obtain a sufficient number of live lymph node cells from culture with APC infected with H5N1 HPAIVs, we analyzed cells from macaques infected with NRT1 (Figure 4B). IFN-γ-positive cells in CD8^+^ ((NR4 (12.6%), NR6 (10.5%)) and CD4^+^ (NR4 (4.2%)) cells cultured with APC infected with NRT1 were detected more frequently than IFN-γ- positive cells in CD8^+^ (NR4 (2.0%), NR6 (7.3%)) and CD4^+^ (NR4 (0.3%)) cells cultured with uninfected APC. In NR5, the percentage of IFN-γ-positive cells in CD4^+^ and CD8^+^ cells did not increase after culture with APC infected with NRT1. These results indicated that CD4^+^ and/or CD8^+^ cells in two macaques vaccinated with Vac-3 were reactive to NRT1-infected cells. Therefore, both humoral and cellular immune responses against NRT1 were detected in macaques vaccinated with Vac-3.

## Discussion

Potency of Vac-3 vaccine was previously examined in chickens using H5N1 HPAIVs [15]. In the present study, to evaluate efficacy of the vaccine in a non-human primate model, we selected challenge strains that propagated vigorously in macaques. Two H5N1 HPAIVs, VN3040 (clade 1) and HOK1 (clade 2.3.2.1), caused viral pneumonia in macaques without vaccination, to degrees similar to that reported previously [19], and with more severe morbidity than did another H5N1 HPAIV, A/Vietnam/1194/2004 (clade 1) [11]. Subcutaneous vaccination with inactivated whole particles of Vac-3 induced anti-HA neutralization antibody against H5N1 HPAIVs after challenge infection and anti-NA antibody with neuraminidase inhibition activity against Vac-3 and pandemic (H1N1) 2009 virus. Vaccination with Vac-3 also activated T cells producing IFN-γ in lymph nodes of macaques. In challenge infection, virus propagation was significantly inhibited in macaques infected with either H5N1 HPAIVs or H1N1 pandemic virus but not in macaques infected with H7N7 HPAIV even when only three macaques (the minimal number for statistics) were used in each group to reduce the number of animals in the study. Therefore, the whole particle vaccine of Vac-3 was effective for preventing propagation of two different clades of H5N1 HPAIVs and a different subtypic H1N1 virus.

Cross-clade immune responses induced by H5N1 influenza vaccines have been reported in mice [9], [20], [21] and ferrets [22], [23]. In a nonhuman primate model, cross-clade neutralizing antibodies induced by an H5N1 influenza vaccine were reported [24], but protective immunity against challenge infection has not been assessed. In humans, results of a clinical trial of a whole particle H5N1 vaccine were reported [25], and cellular responses were evaluated [26]. However, the efficacy of vaccines against challenge infection was unknown due to difficulty in performing challenge infection in humans. Furthermore, according to epidermiologic behavior of influenza viruses in humans, it was believed that natural infection and current vaccination with split vaccines induced only weak cross-reactive immunity in humans [27], [28]. Thus, we evaluated the crossreactivity of immune responses induced by a whole particle H5N1 vaccine in a non-human primate model, in which immunological responses are presumably the closest to those in humans among available experimental animals.

Seven days after challenge infection with H5N1 HPAIVs, all macaques vaccinated with Vac-3 showed neutralization activity in plasma, but neutralization antibody against H5N1 viruses was not detected in plasma of the unvaccinated macaques. Therefore, it was thought that the neutralization activity against challenge viruses was induced by vaccination. Furthermore, neutralization activity against Vac-3 on day 7 after infection was higher than that against Vac-3 on day 0. This means that challenge infection with H5N1 HPAIVs activated B cells specific for Vac-3. Since neutralization activity against challenge strains was not detected before challenge infection, neutralization antibody induced by Vac-3 mainly reacted with Vac-3 before infection, but challenge infection activated B cells producing antibodies reacting with both Vac-3 and H5N1 HPAIV challenge strains. Therefore, the Vac-3 vaccine would elicit antibodies against common antigenic epitopes shared among Vac-3, clade 1, and clade 2.3.2.1 H5N1 viruses.

Inhibition of virus propagation was also observed in the vaccinated macaques infected with NRT1. Since no neutralization activity against NRT1 was detected in plasma of the vaccinated macaques before and after challenge infection with NRT1, immunity other than antibodies against HA would contribute to protection against NRT1 infection. NI activity against Vac-3 and NRT1 was detected in plasma of the vaccinated macaques, and the NI activity in two macaques (NR4 and NR5) was increased after challenge infection. In addition, IFN-γ-producing CD4^+^ and CD8^+^ T cells specific for NRT1 antigens were detected in two vaccinated macaques (NR4 and NR6). Therefore, it was thought that both antibodies against NA and T cells specific for antigens shared in Vac-3 and NRT1 contributed to protection against NRT1 infection in the vaccinated macaques.

The extent of contribution of anti-NA antibodies and T cells to protective immunity in previous studies varied [29]–[32] and was dependent on individual macaques in the present study. In NR4, both anti-NA antibodies and T cells specific for NRT1 antigens seemed to have worked for inhibition of virus propagation since both NI activity and IFN-γ-producing CD8^+^/CD4^+^ T cells against NRT1 were increased after challenge infection. In NR5, anti-NA antibodies might have mainly worked for inhibition of virus propagation rather than T cells since NI activity in plasma was increased after infection, but IFN-γ responses against NRT1 by CD4^+^ and CD8^+^ T cells were not detected. On the other hand, in NR6, CD8^+^ T cells might have mainly worked for protection since IFN-γ-producing CD8^+^ T cells but not CD4^+^ T cells against NRT1 were detected without an increase in NI activity after infection. Although CD8^+^ T cells were thought to be important to eliminate influenza viruses in mice [33] and preexisting influenza-specific CD4^+^ T cells correlated with disease severity in humans [34], the role of T cells, either CD8^+^ or CD4^+^ T cells, in human influenza virus infection remains elusive since both CD4^+^ and CD8^+^ T cells were expanded 7 days after challenge infection [34]. In our nonhuman primate model of influenza virus infection, the differential contribution of T cell subsets in individual macaques suggests that genetic variations including major histocompatibility complex haplotypes might affect immune responses in vaccination and in infection [13].

Vaccination with Vac-3 did not show protection against H7N7 HPAIV infection in macaques. Vaccinated macaques infected with H7N7 HPAIV showed severe morbidity, and virus titers in swab samples from the vaccinated macaques were at similar levels to those in swab samples from the unvaccinated macaques. No neutralization activity in plasma against H7N7 was induced in vaccinated macaques, but IFN-γ production by T lymphocytes against NL2586 was detected. These results suggested that antibody responses with neutralization activity were more important than T lymphocyte responses in protection against HPAIV infection, though T lymphocyte responses against H5N1 HPAIVs might have helped the elimination of VN3040 and HOK1 viruses in vaccinated macaques [35].

In summary, we demonstrated that a whole particle vaccine of Vac-3 induced protective immune responses against two clades of H5N1 HPAIVs and a pandemic (H1N1) 2009 strain in cynomolgus macaques. We found that neutralization activity induced by vaccination was crucial for protection from HPAIVs and that antibody responses with neuraminidase inhibition activity and T lymphocyte responses might be sufficient to protect against non-HPAIV virus infection. In our previous study on a comparison of the efficacy of a whole particle vaccine and that of an ether-split vaccine against pandemic (H1N1) 2009 virus infection [13], protective efficacy of the ether-split vaccine was less potent than that of the whole particle vaccine even when the subtype of the vaccine was the same as that of the challenge strain. Therefore, whole particle vaccines are preferable to induce immunity not only for homosubtypic protection but also for cross-clade and heterosubtypic protection.

## Materials and Methods

### Ethics Statement

This study was carried out in strict accordance with the Guidelines for the Husbandry and Management of Laboratory Animals of the Research Center for Animal Life Science at Shiga University of Medical Science and Standards Relating to the Care and Management, etc. of Experimental Animals (Notification No.6, March 27, 1980 of the Prime Minister’s Office, Japan). The protocols were approved by the Shiga University of Medical Science Animal Experiment Committee (Permit numbers: 2008-5-11, 2009-8-6H, 2010-10-1, 2011-4-2H, and 2012-10-8). The animal experiments were conducted in strict compliance with animal husbandry and welfare regulations. The Research Center for Animal Life Science at the Shiga University of Medical Science has a permit for importation of cynomolgus macaques. Regular veterinary care and monitoring, balanced nutrition and environmental enrichment were provided by the Research Center for Animal Life Science at the Shiga University of Medical Science. The macaques were euthanized at endpoint (7 days after virus inoculation) using ketamine/xylazine followed by intravenous injection of pentobarbital (200 mg/kg). Animals were monitored every day during the study to be clinically scored as shown in Table S4 [19] and to undergo veterinary examinations to help alleviate suffering. Animals would be euthanized if their clinical scores reached 15 (a humane endpoint).

### Animals

Five- to eight-year-old female cynomolgus macaques from Vietnam and the Philippines were used. The cynomolgus macaques used in the present study were healthy adults. All procedures were performed under ketamine and xylazine anesthesia, and all efforts were made to minimize suffering. Food pellets of CMK-2 (CLEA Japan, Inc., Tokyo, Japan) were provided once a day after recovery from anesthesia and drinking water was available *ad libitum.* Animals were singly housed in the cages equipping bars to climb up and puzzle feeders for environmental enrichment under controlled conditions of humidity (26–64%), temperature (24–26°C), and light (12-h light/12-h dark cycle, lights on at 8∶00 A.M.). In the text and figures, individual macaques are distinguished by identification numbers (Table S1). The absence of influenza A virus NP-specific antibodies in their plasma was confirmed before experiments using an antigen-specific enzyme-linked immunosorbent assay (ELISA), AniGen AIV Ab ELISA (Animal Genetics Inc., Kyonggi-do, Korea), for currently circulating influenza virus. Two weeks before virus inoculation, a telemetry probe (TA10CTA-D70, Data Sciences International, St. Paul, MN) was implanted in the peritoneal cavity of each macaque under ketamine/xylazine anesthesia followed by isoflurane inhalation to monitor body temperature. The macaques used in this study were free from herpes B virus, hepatitis E virus, *Mycobacterium tuberculosis*, *Shigella* spp., *Salmonella* spp., and *Entamoeba histolytica*.

Under ketamine/xylazine anesthesia, two cotton sticks (TE8201, Eiken Chemical, Ltd., Tokyo, Japan) were used to collect fluid samples in nasal cavities, oral cavities, and tracheas, and the sticks were subsequently immersed in 1 ml of PBS containing 0.1% bovine serum albumin (BSA) and antibiotics. A bronchoscope (MEV-2560, Machida Endoscope Co. Ltd., Tokyo, Japan) and cytology brushes (BC-203D-2006, Olympus Corporation, Tokyo, Japan) were used to obtain bronchial samples [36].

### Viruses and Vaccines

We used the influenza virus A/duck/Hokkaido/Vac-3/2007 (H5N1) (Vac-3, National Center for Biotechnology Information (NCBI) taxonomy database ID: 463698) as a vaccine strain [15]. This virus is a reassortant virus between A/duck/Hokkaido/101/2004 (H5N3) and A/duck/Hokkaido/262/2004 (H6N1) [14].

The Vac-3 virus was propagated in allantoic cavities of 10-day-old embryonated hen’s eggs at 35°C for 48 h. To prepare an inactivated whole particle vaccine, the allantoic fluid infected with Vac-3 was concentrated and purified by high-speed centrifugation (112,500 g for 90 min) through a 10–50% sucrose density gradient and then treated in 0.2% formalin at 4°C for one week [15]. Inactivation of viruses in the vaccine was confirmed by the absence of detectable hemagglutination activity following inoculation of the materials into embryonated eggs after one passage. The amount of the whole particle vaccine is indicated as the amount of entire proteins including HA and other viral proteins. The whole particle vaccine (1 mg/dose) was inoculated subcutaneously into macaques using syringes twice with a two-week interval between injections under ketamine/xylazine anesthesia.

The following virus strains were used as challenge strains: A/Vietnam/UT3040/2004 (H5N1) (VN3040, NCBI taxonomy ID: 755291) isolated from a human patient [37], [38], A/whooper swan/Hokkaido/1/2008 (H5N1) (HOK1, NCBI taxonomy ID: 527168) isolated from a dead swan [39], and A/chicken/Netherlands/2586/2003 (H7N7) (NL2586, NCBI taxonomy ID: 533037, kindly provided by Dr. I. Capua (L’Office International des Épizooties (OIE), Food and Agriculture Organization of the United Nations (FAO), and National Reference Laboratory for Newcastle Disease and Avian Influenza, Istituto Zooprofilattico Sperimentale delle Venezie, Italy) [12], [16]. These viruses were highly pathogenic avian influenza viruses. A/Narita/1/2009 (H1N1) (NRT1, NCBI taxonomy ID: 645520, kindly provided by Dr. Takato Odagiri, National Institute of Infectious Disease (NIID), Japan) [40] was a pandemic influenza virus. VN3040 was propagated twice in Madin-Darby canine kidney (MDCK) cells at the University of Tokyo. HOK1 and NL2586 were propagated twice in embryonated eggs. NRT1 was propagated in embryonated eggs twice at NIID and once in MDCK cells (the American Type Culture Collection, Manassas, VA) at the Shiga University of Medical Science [13].

The macaques were challenged with VN3040 (3×10^6^ PFU_50_/7 ml), HOK1 (3×10^6^ PFU/7 ml), and NRT1 (3×10^6^ TCID_50_/7 ml) inoculated into the nostrils (0.5 ml for each nostril), oral cavity (0.5 ml on the surface of each tonsil), and trachea (5 ml) with pipettes and catheters 5 weeks after the second vaccination under ketamine/xylazine anesthesia [17]. NL2586 (4×10^7^ TCID_50_/2 ml) was inoculated into the nostrils (0.45 ml for each nostril), conjunctiva (0.05 ml for the surface of each conjunctiva), and trachea (1 ml) [16]. In the present study, inoculation of NRT1 and NL2586 in unvaccinated macaques was not performed, but results were cited from the previous studies [16], [17]. The identical lots and titers of NRT1 and NL2586 as those used in the previous studies were inoculated into vaccinated macaques in the present study. The inoculation volumes of NRT1 and NL2586 in vaccinated macaques were determined in accordance with the volumes in unvaccinated macaques in the previous report [16], [17]. Experiments using challenge virus were performed in the biosafety level 3 facility of the Research Center for Animal Life Science, Shiga University of Medical Science.

In order to assess virus replication, serial dilutions of swab samples were inoculated onto confluent MDCK cells as described previously [11]. Cytopathic effects were examined under a microscope 72 h later.

### Detection of Antibody Specific for Virus Antigen by ELISA

The antibody titers of plasma and swab samples against Vac-3 antigens were determined using ELISA [41]. Ninety-six-well plates were coated with 50 µl of purified Vac-3 (20 µg/ml) disrupted with 0.05 M Tris-HCl (pH 7.8) containing 0.5% Triton X-100 and 0.6 M KCl. Serially diluted samples were incubated overnight in the coated plates. After washing five times, horseradish peroxidase (HRP)–conjugated anti-monkey IgG antibodies (MP Biomedicals, Inc./Cappel, Aurora, OH) (1∶2000×50 µl) and anti-monkey IgA antibodies (Nordic Immunological Laboratories, Tilburg, The Netherlands) (1∶4000×50 µl) were added and incubated for 1 h at room temperature. HRP activity was assessed using 3, 3′, 5, 5′-tetramethyl benzidine substrate (100 µl). The reaction was stopped by the addition of 1 M hydrogen chloride (100 µl). Optical density (OD) was measured at 450 nm and 620 nm. Results are shown after subtraction of OD at 620 nm from OD at 450 nm.

### Virus Neutralization Assay

Plasma samples were pretreated with a receptor-destroying enzyme (RDEII, Denka Seiken, Tokyo, Japan) at 37°C overnight and then inactivated at 56°C for 1 h. The diluted samples were mixed with 50 TCID_50_ of the VN3040, HOK1, NL2586, or NRT1 viruses for 1 h. Then the mixture was added onto an MDCK monolayer. After 1-h incubation, the cells were cultured in Eagle’s minimal essential medium (MEM) containing 0.1% BSA. For virus propagation of NRT1, 5 µg/ml trypsin was added to the medium. After incubation at 35°C for 3 days, the number of wells with cytopathic effects was counted in quadruplicate culture. Neutralization titers were expressed as the dilution in which cytopathic effects were observed in 50% of the wells.

For neutralization of Vac-3, 200 PFU of Vac-3 were mixed with the diluted samples for 1 h. Then the mixture was added onto the MDCK monolayer in 6-well plates. After 1 h, MDCK cells were covered by MEM containing 1% agar, 0.1% BSA, and 5 µg/ml trypsin. After incubation at 35°C for 2 days, the number of plaques was counted. Neutralization titers were expressed as the dilution in which the numbers of plaques were reduced to 50% of plaques without plasma.

### Neuraminidase Inhibition Test

Diluted plasma (25 µl) was incubated at room temperature for 1 h with Vac-3 or NRT1 virus. The dilution of the virus that was used in the assays was standardized to the amount of NA activity providing 0.5 OD at 549 nm in the thiobarbituric acid (TBA) assay. Fetuin (50 µl, 25 mg/ml in 3 mM CaCl_2_•6H_2_O, 80 mM Na_2_HPO_4_, 300 mM NaH_2_PO_4_, pH 5.9) was added and incubated for 16 h at 37°C. NaIO_4_ (50 µl, 4.28 g in phosphate 62 ml+H_2_O 38 ml) was mixed for 20 min at room temperature. NaAsO_2_ solution (0.5 ml, 10 g NaAsO_2_ and 7.1 g Na_2_SO_4_ in 100 ml H_2_O+0.3 ml H_2_SO_4_) was added and mixed with a vortex. TBA solution (1.25 ml, TBA 6 mg/ml+Na_2_SO_4_ 71 mg/ml) was added to samples, boiled for 15 min, and then cooled on ice. N-butanol (1.5 ml) was added, mixed with a vortex, and spun down for 10 min at 4000 rpm. ODs at 549 nm of upper layers were measured. The NI titers were determined as the reciprocal of the highest dilution of plasma that caused 50% inhibition of NA activity.

### B Cell Lines

Blood cells were obtained before the first vaccination. Lymphocytes were purified from peripheral blood of the macaques using a density gradient (Wako Pure Chemical Industries Ltd., Osaka, Japan). After washing, cells were cultured in RPMI-1640 supplemented with 12% fetal calf serum (FCS) and supernatants of TsB-B6 cells producing herpesvirus macaca fascicularis I (kindly provided by Dr. Kazuhiko Hayashi, Tottori University, Japan) [42], [43]. More than 90% of the cells used in the present study were positive for CD20.

Transformed B cells were washed twice with Hanks buffered salt solution, and then influenza virus was added. After 30-min incubation at 35°C, the cells were cultured in 5 ml of RPMI-1640 supplemented with 10% FCS and 2-mercaptoethanol (ME) overnight. Mitomycin C (5 µg/ml) was added for the last 30 min. Cells were washed four times with Hanks buffered salt solution and thereafter used as antigen-presenting cells.

### Detection of IFN-γ Production by Lymph Node Cells

Unfractionated cells (5×10^5^/well) from cervical lymph nodes obtained at autopsy on day 4 or 7 after the challenge infection were incubated with autologous B cell lines (2.5×10^5^/well) in the presence of anti-CD28 (clone: CD28.2) and anti-CD49d (clone: 9F10) antibodies (0.5 µg/ml, eBioscience Inc., San Diego, CA) in 96-well U-bottom plates for 2 d. The concentration of IFN-γ in the supernatants was measured using Panomix Procarta Immunoassays for non-human primate cytokines (Affymetrix, Inc., Santa Clara, CA) and Luminex200 (Millipore Corp., Billerica, MA).

### Flow Cytometry for IFN-γ Detection

Unfractionated cells (5×10^5^/well) from cervical lymph nodes obtained at autopsy were incubated with autologous B cell lines (2.5×10^5^/well) in the presence of anti-CD28, anti-CD49d antibodies (0.5 µg/ml), and human IL-2 (10 ng/ml, Miltenyi Biotec GmbH, Bergisch Gladbach, Germany) in 96-well U-bottom plates for 5 d. The cultured lymph node cells were stimulated with phorbol 12-myristate 13-acetate (PMA) (0.1 µg/ml) and ionomycin (1 µg/ml) for 5 h. Monensin (2 µM) was added for the last 4 h. Thereafter, cells were washed with PBS containing EDTA (0.5 µM). Surface CD8 and CD4 were stained with fluorescein isothiocyanate (FITC)- and allophycocyanin (APC)-conjugated specific antibodies (clones: RPA-T8 and OKT-4, eBioscience), respectively. Intracellular IFN-γ was stained with phycoerythrin (PE)-conjugated antibody (clone: 4S.B3, eBioscience) after fixation with 4% paraformaldehyde and permeabilization with 0.1% saponin. Dead cells were excluded by staining with ethidium monoacetate (EMA, Molecular Probes, Inc., Eugene, OR). IFN-γ-positive cells were calculated as the percentage in CD8- and CD4-positive cells [13].

### Histopathological Examination and Detection of Viral Antigen by Immunofluorescence Staining

For histopathological examination, lungs obtained at autopsy were immersed in 10% neutral buffered formalin for fixation, embedded in paraffin, and cut to 3 µm thick on glass slides. Sections were stained with hematoxylin and eosin (H & E) and observed under a light microscope. For immunofluorescence staining, lung tissues were embedded in O.C.T. compound, frozen, and cut to 5 µm thick on glass slides. After acetone fixation, sections were incubated with 1% BSA in PBS for 30 min and double stained with eFluor615-conjugated anti-pan-cytokeratin antibody (1∶50 dilution, clone: AE1/AE3, eBioscience) and FITC-conjugated goat polyclonal anti-influenza A virus antibody (1∶25 dilution, Abcam plc, Cambridge, United Kingdom) for 1 h at room temperature. Nuclei were counterstained with 4′, 6-diamidino-2-phenylindole (DAPI). Then the tissues were observed under a fluorescent microscope (FSX-100, Olympus Corporation). The image data were analyzed with the cellSens Dimension Desktop 1.5 software (Olympus Corporation).

## Supporting Information

Figure S1 **Clinical scores and total virus titers in challenge infection.** (A–D) Clinical scores were determined by daily observation and body temperature, according to Table S4. Averages and standard deviations of clinical scores are shown in each group. (E–H) Virus titer areas under the virus titer time curves (virus titer AUC) in nasal, oral, tracheal, and bronchial samples were calculated on the basis of titers in Tables 1, 2, 4, and 5. Virus titers under the detection limit are calculated as 0. Averages and standard deviations of three macaques are shown in each group. Therefore, results of virus titers until day 7 (E and H), day 5 (F), and day 4 (G) are used for calculation. A, E: macaques infected with VN3040; B, F: macaques infected with HOK1; C, G: macaques infected with NL2586; D, H: macaques infected with NRT1. Red lines and bars: unvaccinated macaques, blue lines and bars: vaccinated macaques. Asterisks indicate significant differences between unvaccinated groups and vaccinated groups (P<0.05, Student’s t-test).
(TIF)

Figure S2 **Antibody responses specific for Vac-3 antigens in vaccinated cynomolgus macaques.** Cynomolgus macaques were subcutaneously immunized twice (in weeks 0 and 2) with a whole virus particle vaccine derived from Vac-3. Plasma and swab samples were collected in indicated weeks after the first vaccination. Lines indicate results of individual macaques. IgG (A–C) and IgA (D–F) antibodies specific for Vac-3 antigens in plasma (A, D), nasal swab samples (B, E), and tracheal swab samples (C, F) were analyzed using ELISA. Optical densities at 450 nm at indicated dilution are shown.
(TIF)

Figure S3 **Body temperature of unvaccinated and vaccinated macaques infected with H7N7 highly pathogenic avian influenza virus or pandemic (H1N1) 2009 virus.** H7N7 highly pathogenic avian influenza virus (NL2586) (upper) or pandemic (H1N1) 2009 virus (NRT1) (lower) was inoculated on day 0 (five weeks after the second vaccination) (right). Body temperature of unvaccinated macaques was reanalyzed and cited from the previous studies for comparison (left) [16], [17]. Body temperatures of macaques were recorded using telemetry transmitters and a computer. Temperatures from 6 P.M. to 10 A.M. are shown in the graphs since temperatures between 10 A.M. and 6 P.M. were affected by anesthesia.
(TIF)

Table S1 **Cynomolgus macaques used in the present study.** Abbreviations of challenge virus strains are used in the text and figures. Unvaccinated (#1–#3) and vaccinated monkeys (#4–#6) were used in this study.
(PDF)

Table S2 **Virus titers in tissues obtained at autopsy.** Highly pathogenic avian influenza virus A/Vietnam/UT3040/2004 (H5N1) (VN3040) or A/whooper swan/Hokkaido/1/2008 (H5N1) (HOK1) was inoculated into the nostrils, oral cavity, and trachea of each macaque on day 0. VN1, VN2, VN3, Ho1, and Ho2 were autopsied 7 days after virus inoculation. The dead macaque Ho3 was autopsied 5 days after virus infection. Tissue pieces of indicated organs were collected and virus titers in the tissues were determined. <: Virus titers under the detection limit (<1.67 TCID_50_/g tissue). R: right, L: left, RU: right upper lobe, RM: right middle lobe, RL: right lower lobe, LU: left upper lobe, LM: left middle lobe, LL: left lower lobe, LN: lymph nodes.
(PDF)

Table S3 **Similarity of amino acid sequences in HA and NA between Vac-3 and challenge strains.** Amino acid sequences of challenge strains are compared with that of a vaccine strain, Vac-3. GI numbers of HA and NA were assigned by the NCBI.
(PDF)

Table S4 **Clinical scoring used in this study.** Animals were monitored every day during the study to be clinically scored. Animals would be euthanized if their clinical scores reached 15 (a humane endpoint).
(PDF)
