# Supplementary figures and images for: Protection against H5N1 Highly Pathogenic Avian and Pandemic (H1N1) 2009 Influenza Virus Infection in Cynomolgus Monkeys by an Inactivated H5N1 Whole Particle Vaccine
Source: PLoS One. 2013 Dec 23;8(12):e82740. doi: 10.1371/journal.pone.0082740 (PMC3871535; doi:10.1371/journal.pone.0082740)

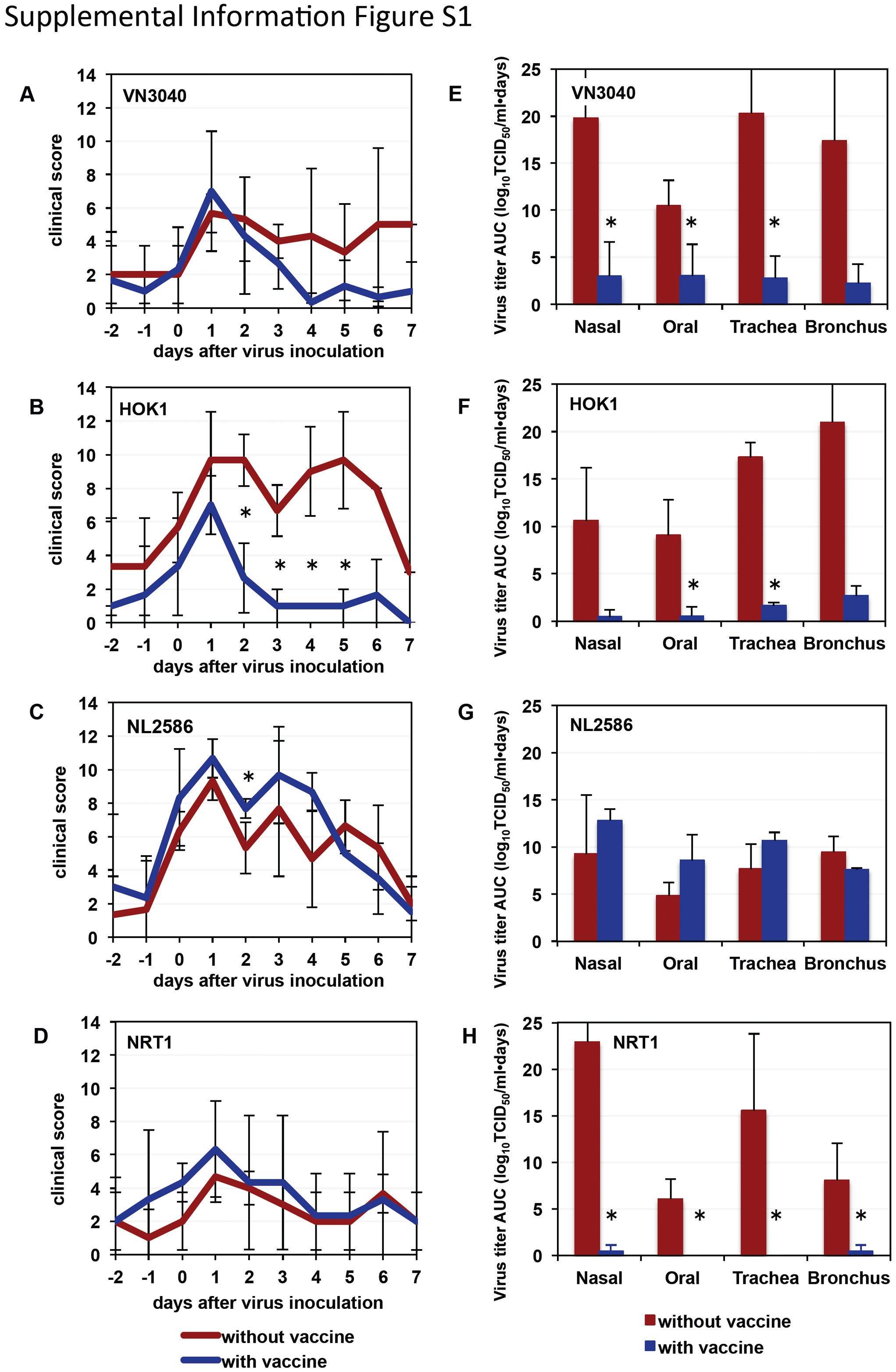

Supplement: Figure S1 — Clinical scores and total virus titers in challenge infection. (A–D) Clinical scores were determined by daily observation and body temperature, according to Table S4. Averages and standard deviations of clinical scores are shown in each group. (E–H) Virus titer areas under the virus titer time curves (virus titer AUC) in nasal, oral, tracheal, and bronchial samples were calculated on the basis of titers in Tables 1, 2, 4, and 5. Virus titers under the detection limit are calculated as 0. Averages and standard deviations of three macaques are shown in each group. Therefore, results of virus titers until day 7 (E and H), day 5 (F), and day 4 (G) are used for calculation. A, E: macaques infected with VN3040; B, F: macaques infected with HOK1; C, G: macaques infected with NL2586; D, H: macaques infected with NRT1. Red lines and bars: unvaccinated macaques, blue lines and bars: vaccinated macaques. Asterisks indicate significant differences between unvaccinated groups and vaccinated groups (P<0.05, Student’s t-test). (TIF) [file pone.0082740.s001.tif]

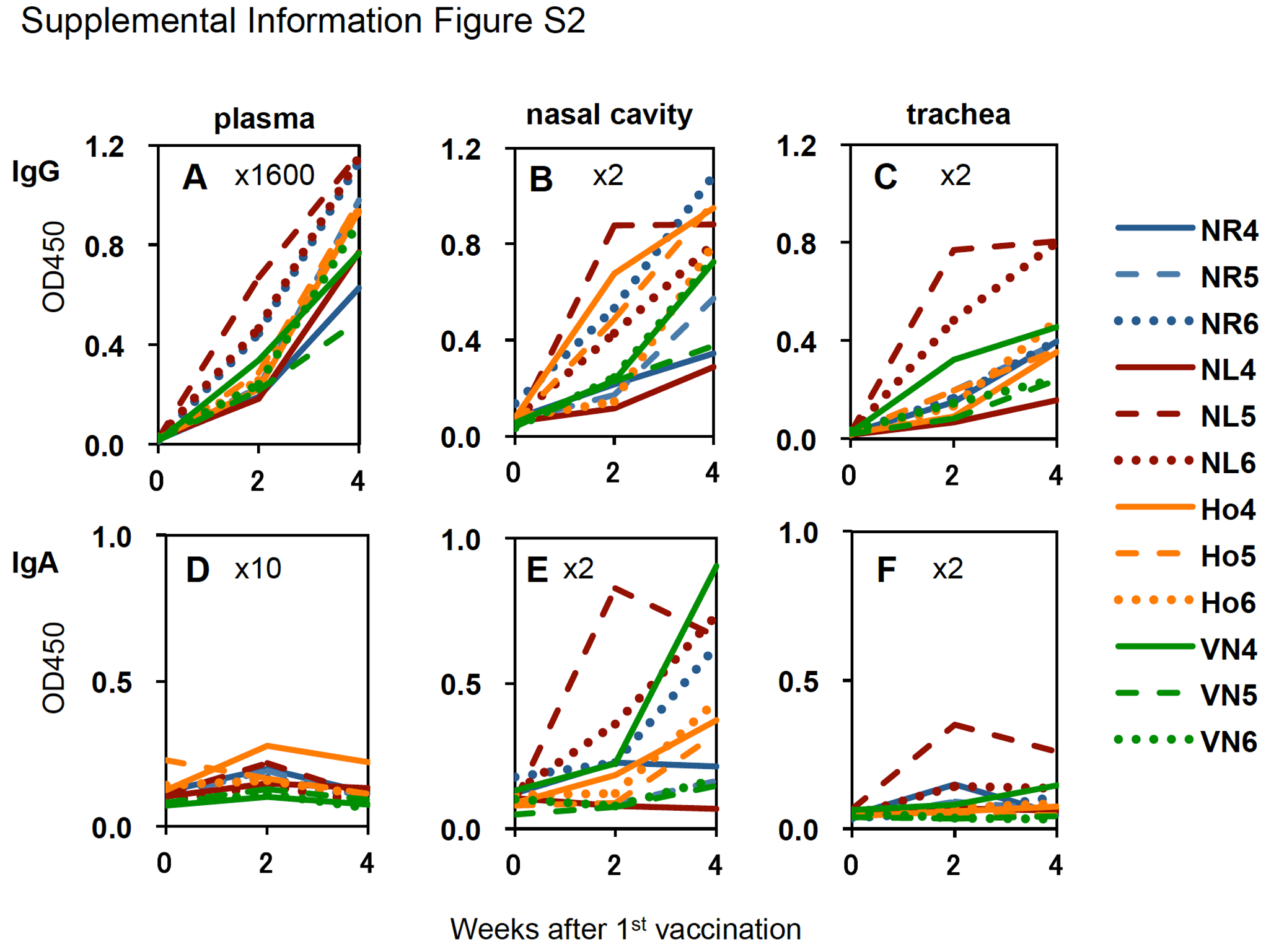

Supplement: Figure S2 — Antibody responses specific for Vac-3 antigens in vaccinated cynomolgus macaques. Cynomolgus macaques were subcutaneously immunized twice (in weeks 0 and 2) with a whole virus particle vaccine derived from Vac-3. Plasma and swab samples were collected in indicated weeks after the first vaccination. Lines indicate results of individual macaques. IgG (A–C) and IgA (D–F) antibodies specific for Vac-3 antigens in plasma (A, D), nasal swab samples (B, E), and tracheal swab samples (C, F) were analyzed using ELISA. Optical densities at 450 nm at indicated dilution are shown. (TIF) [file pone.0082740.s002.tif]

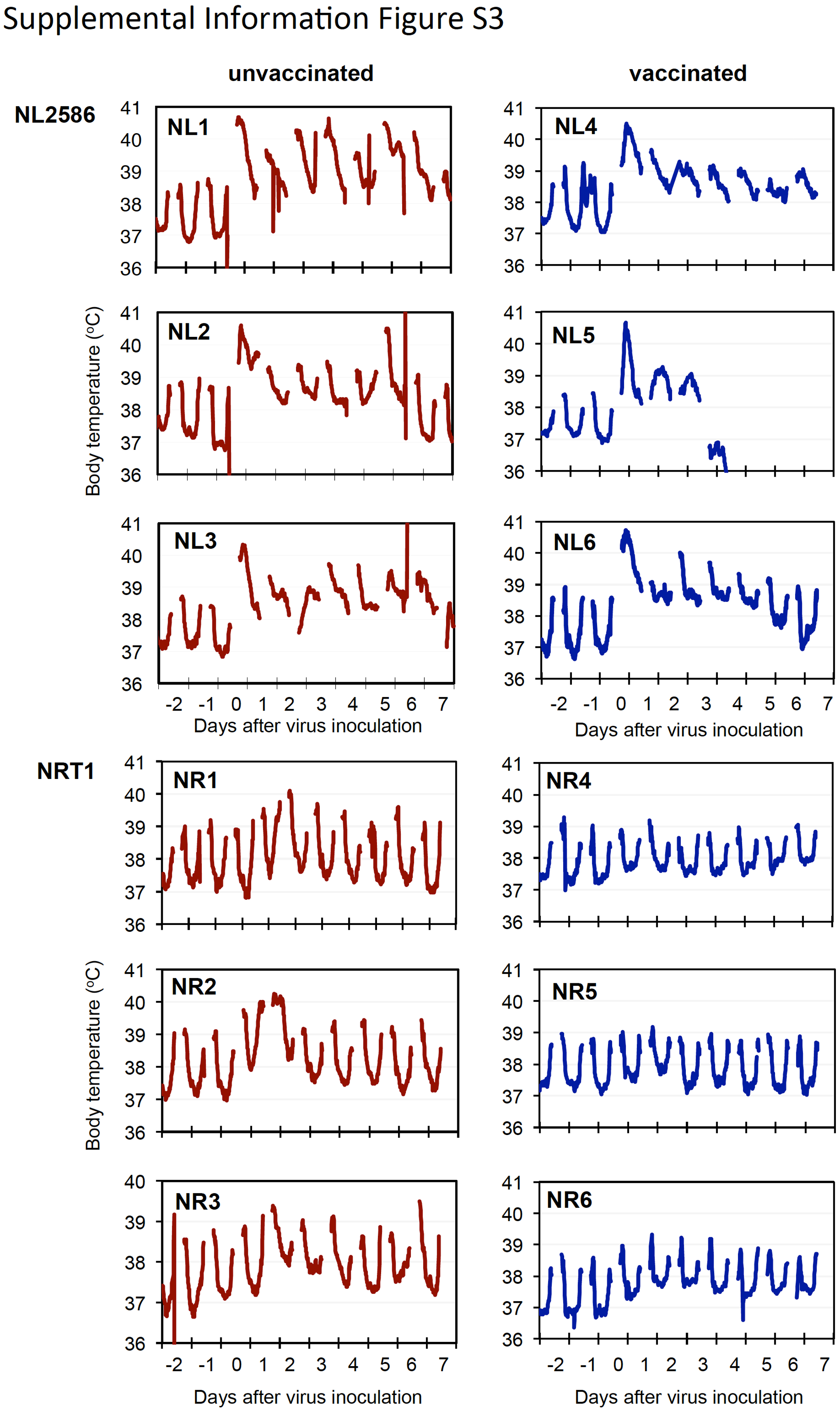

Supplement: Figure S3 — Body temperature of unvaccinated and vaccinated macaques infected with H7N7 highly pathogenic avian influenza virus or pandemic (H1N1) 2009 virus. H7N7 highly pathogenic avian influenza virus (NL2586) (upper) or pandemic (H1N1) 2009 virus (NRT1) (lower) was inoculated on day 0 (five weeks after the second vaccination) (right). Body temperature of unvaccinated macaques was reanalyzed and cited from the previous studies for comparison (left) [16], [17]. Body temperatures of macaques were recorded using telemetry transmitters and a computer. Temperatures from 6 P.M. to 10 A.M. are shown in the graphs since temperatures between 10 A.M. and 6 P.M. were affected by anesthesia. (TIF) [file pone.0082740.s003.tif]
