# Supplementary material for: Protection against H5N1 Highly Pathogenic Avian and Pandemic (H1N1) 2009 Influenza Virus Infection in Cynomolgus Monkeys by an Inactivated H5N1 Whole Particle Vaccine
Source: PLoS One. 2013 Dec 23;8(12):e82740. doi: 10.1371/journal.pone.0082740 (PMC3871535; doi:10.1371/journal.pone.0082740)
Supplement: Table S1 — Cynomolgus macaques used in the present study. Abbreviations of challenge virus strains are used in the text and figures. Unvaccinated (#1–#3) and vaccinated monkeys (#4–#6) were used in this study. (PDF) [file pone.0082740.s004.pdf]

Table S1. Cynomolgus macaques used in the present study

Challenge virus strain	Abbreviation	Without vaccination	With vaccination
A/Vietnam/UT3040/2004 (H5N1)	VN3040	VN1, VN2, VN3	VN4, VN5, VN6
A/whooper swan/Hokkaido/1/2008 (H5N1)	HOK1	Ho1, Ho2, Ho3	Ho4, Ho5, Ho6
A/chicken/Netherlands/2586/2003 (H7N7)	NL2586	NL1, NL2, NL3	NL4, NL5, NL6
A/Narita/1/2009 (H1N1)	NRT1	NR1, NR2, NR3	NR4, NR5, NR6
