# Supplementary material for: Protection against H5N1 Highly Pathogenic Avian and Pandemic (H1N1) 2009 Influenza Virus Infection in Cynomolgus Monkeys by an Inactivated H5N1 Whole Particle Vaccine
Source: PLoS One. 2013 Dec 23;8(12):e82740. doi: 10.1371/journal.pone.0082740 (PMC3871535; doi:10.1371/journal.pone.0082740)
Supplement: Table S2 — Virus titers in tissues obtained at autopsy. Highly pathogenic avian influenza virus A/Vietnam/UT3040/2004 (H5N1) (VN3040) or A/whooper swan/Hokkaido/1/2008 (H5N1) (HOK1) was inoculated into the nostrils, oral cavity, and trachea of each macaque on day 0. VN1, VN2, VN3, Ho1, and Ho2 were autopsied 7 days after virus inoculation. The dead macaque Ho3 was autopsied 5 days after virus infection. Tissue pieces of indicated organs were collected and virus titers in the tissues were determined. <: Virus titers under the detection limit (<1.67 TCID50/g tissue). R: right, L: left, RU: right upper lobe, RM: right middle lobe, RL: right lower lobe, LU: left upper lobe, LM: left middle lobe, LL: left lower lobe, LN: lymph nodes. (PDF) [file pone.0082740.s005.pdf]

Table S2. Virus titers in tissues obtained at autopsy

Virus	VN3040			HOK1		
Animal	VN1	VN2	VN3	Ho1	Ho2	Ho3
Tissue/Autopsy day	7	7	7	7	7	5
Oro/nasopharynx	<	<	<	4.00	2.50	4.50
Tonsil R	4.33	1.67	2.33	2.67	3.67	3.50
Tonsil L	4.33	<	3.67	1.67	1.67	2.67
Trachea	4.00	1.67	<	2.50	3.67	5.33
Bronchus R	1.67	<	<	3.50	3.67	4.50
Bronchus L	3.67	<	<	3.33	3.37	4.23
Lung RU	3.67	<	<	3.33	3.50	5.67
Lung RM	3.33	<	1.67	4.00	4.50	5.00
Lung RL	4.67	<	<	2.67	5.00	5.50
Lung LU	4.23	<	<	1.67	4.67	5.33
Lung LM	1.67	<	<	2.00	4.50	5.50
Lung LL	1.67	<	<	4.33	4.67	4.50
Mediastinal LN	1.67	<	<	<	<	2.67
Cerebrum frontal lobe	<	<	<	<	<	2.67
Muscle	<	<	<	<	<	4.50
Jejunum	<	1.67	<	<	<	<

<: Virus titers under the detection limit (< 1.67 $TCID_{50}$/g tissue). R: right, L: left, RU: right upper lobe, RM: right middle lobe, RL: right lower lobe, LU: left upper lobe, LM: left middle lobe, LL: left lower lobe, LN: lymph nodes.
