# Supplementary material for: Protection against H5N1 Highly Pathogenic Avian and Pandemic (H1N1) 2009 Influenza Virus Infection in Cynomolgus Monkeys by an Inactivated H5N1 Whole Particle Vaccine
Source: PLoS One. 2013 Dec 23;8(12):e82740. doi: 10.1371/journal.pone.0082740 (PMC3871535; doi:10.1371/journal.pone.0082740)
Supplement: Table S3 — Similarity of amino acid sequences in HA and NA between Vac-3 and challenge strains. Amino acid sequences of challenge strains are compared with that of a vaccine strain, Vac-3. GI numbers of HA and NA were assigned by the NCBI. (PDF) [file pone.0082740.s006.pdf]

Table S3. Similarity of amino acid sequences in HA and NA between Vac-3 and challenge strains.

		HA		NA	
Strain	Subtype	Similarity (%)	GI number	Similarity (%)	GI number
Vac-3	H5N1	-	156522879	-	156522883
VN3040	H5N1	93	295189630	90	295189542
HOK1	H5N1	91	188035684	89	188035688
NL2586	H7N7	43	189178851	43	189178855
NRT1	H1N1	66	237659680	90	237681746
