# Supplementary material for: Protection against H5N1 Highly Pathogenic Avian and Pandemic (H1N1) 2009 Influenza Virus Infection in Cynomolgus Monkeys by an Inactivated H5N1 Whole Particle Vaccine
Source: PLoS One. 2013 Dec 23;8(12):e82740. doi: 10.1371/journal.pone.0082740 (PMC3871535; doi:10.1371/journal.pone.0082740)
Supplement: Table S4 — Clinical scoring used in this study. Animals were monitored every day during the study to be clinically scored. Animals would be euthanized if their clinical scores reached 15 (a humane endpoint). (PDF) [file pone.0082740.s007.pdf]

Table S4. Clinical scoring used in this study

Parameter	Degree of parameter	Possible score
Fever	Normal (< 39 ^{o}C)	0
	Elevated temperature (39-40 ^{o}C)	3
	High temperature (> 40 ^{o}C)	5
Posture	Piloerection of body hair	1
	Decreased activity, decreasing normal behavior/Occasionally lying down, huddled, active when people in room	2
	Huddled on camera, active when people in room/Lying down, getting up when approached, using cage for support	3
	Huddled when people in room, shaking, toes and hands clenched/Lying down, not getting up when approached or prompted	5
Respiration	Increased or decreased; mild cough and clear nasal discharge	3
	Labored breathing through mouth; severe cough and severe nasal discharge	5
Appetite	Slightly decreased	1
	Decreased	2
	Severely decreased	5
Skin	Flushed appearance	2
	Visible rash	2
	Bleeding	5
